# Newborn Screening for Congenital Hypothyroidism-Clinical Evaluation and Comparison of Two Different Test Kits for the Determination of TSH in Dried Blood Samples on Two Different Platforms

**DOI:** 10.3390/ijns7030051

**Published:** 2021-08-03

**Authors:** Ralph Fingerhut

**Affiliations:** Swiss Newborn Screening Laboratory, University Children’s Hospital, 8032 Zurich, Switzerland; Ralph.Fingerhut@synlab.com; Tel.: +49-961-309-327; Fax: +49-961-309-224

**Keywords:** congenital hypothyroidism (CH), newborn screening (NBS), thyroid stimulating hormone (TSH), dried blood sample (DBS)

## Abstract

Newborn screening (NBS) for congenital hypothyroidism (CH) started in the 1970s, with the introduction of radioimmuno assays (RIA) for the measurement of thyroxine (T4), and thyroid stimulating hormone (TSH). With the development of sensitive enzyme immune assays (EIA, FIA, FEIA), RIAs were replaced in the newborn screening laboratories. With the increasing number of analytes and centralization of NBS, there is a growing demand of total automation. In the course of method validation, two fully automated platforms for the determination of TSH in dried blood samples (DBS) were compared. The GSP from PerkinElmer (PE), and the NS2400 from Labsystems (LDx), together with the recommended test kits from both manufacturers. Both systems showed good performance, with recoveries, of 103.0% (LDx) and 98.5% (PE), and CVs for intra and interassay variations at various concentrations, between 4.3 and 15.7. Both assays had a good correlation (r^2^ = 0.8814). With LDx/NS2400 platform, TSH values were in the mean 2.09 mU/L higher; however, the difference of both results from the mean was within ±2 SD, up to 30 mU/L, and only for values above 50 mU/L did the difference become bigger. However, this has no influence on the clinical interpretation. No false negative results were observed with either of the two platforms. TSH results obtained with the LDx/NS2400 were slightly higher than those obtained with the PE/GSP; however, the recall rate was lower: 0.059% compared to 0.063%. This can be explained by the much narrower distribution of TSH values. In conclusion, both platforms are equally suitable for medium and large NBS laboratories. However, due to the more open structure the LDx/NS2400 platform has a lot of advantages compared to the totally closed PE/GSP platform.

## 1. Introduction

Newborn screening (NBS) for congenital hypothyroidism (CH) became feasible after the development of radioimmunoassays (RIA) for the measurement of thyroxine (T4) [1] and thyroid stimulating hormone (TSH) from dried blood spots (DBS) [2] in the mid 1970s. Three possible screening strategies for CH were, therefore, possible: Measurement of serum TSH and T4 from cord blood, and measurement of T4 or TSH from a DBS taken at 3–5 days of life [3]. While NBS for CH based on cord blood, could not be centralized, it never got very common. In contrast, TSH or T4 determination from DBS, could be easily integrated into already existing NBS programs for PKU and other metabolic diseases. The pros and cons of these two different approaches have been described, for example by Larsson et al. [4,5]. For further review see [6]. In Europe, except Malta and the Netherlands, where TSH and T4 is measured simultaneously, all other countries have TSH as the primary and decision-making analyte [7]. The situation in the US is different. In total, 43% of the US NBS laboratories use TSH as the primary marker, and only 6 of these do secondary T4 measurement, when TSH is elevated. 39% do it the other way round: First measuring T4 and secondary TSH in case of abnormal T4. Furthermore, 18% measure simultaneously TSH and T4 [8]. In the 1980s, radioimmuno assays were replaced by methods with non-radioactive tracers, such as Eu^3+^, with a time resolved fluorescence measurement [9,10], or enzyme-linked antibodies in enzyme immune assays (EIA, FIA, FEIA). However, the analytical sensitivity is determined by a large number of variables, where the type of tracer is just one. Other variables/factors include instrumental background and noise, non-specific binding, and the specificity of the antibodies used in the respective test kits, which influence the sensitivity via the basic binding equations [11]. Therefore, it is always necessary to compare the performance of different test kits and test platforms, in order to determine and evaluate the sensitivity and reliability of the chosen combination of test kit and test platform. This difference has already previously been reported, even with the test kits from the same manufacturer [12]. This study was performed to evaluate and compare the performance of two different test kits for the determination of TSH from DBS for newborn screening, on two fully automated systems, the genetic screening processor (GSP^TM^) from PerkinElmer, and the NS2400 from Labsystems. Besides the test kits for TSH, for both platforms there are also test kits available for total tyroxine (T4), 17-hydroxyprogesterone (17-OHP), immunoreactive trypsinogene (IRT), biotinidase, galactose-1-phosphate uridyl transferase (GALT), total galactose, glucose-6-phosphate dehydrogenase (G6PD), and phenylalanine. Both systems can handle up to 25 microtiter plates at a time, with the option of continuous loading. The processing time for the different assays is also comparable, since it is mainly determined by the incubation times. Liquid handling is slightly faster on the LDx/NS2400, because it uses 8-channel pipetting technology without additional plastic pipetting tips, while the PE/GSP just uses two pipettes, one for low volumes, and one for high volumes. The major difference between the two systems is the open architecture of the LDx/NS2400. It allows to use all included components (disk remover, washer, fluorimeter/photometer) also as stand-alone instruments, and, in addition, it also allows the use of organic solvents on the instrument. Neither are possible with the PE/GSP. In addition, the LDx/NS2400 is able to transfer liquid from one microtiter plate to another, which increases the flexibility, and prevents floating disk problems with the enzymatic assays [13].

## 2. Materials and Methods

Capillary blood is taken by heel prick between 72 and 96 h of life, and dried on special blood collection devices (903 grade Whatman Filter Paper, or PerkinElmer 226) the so called dried blood spot (DBS) samples. TSH was measured from 3.2 mm punches from the DBS with two different test kits, in parallel, by using the GSP Neonatal hTSH kit on the GSP instrument from PerkinElmer, Turku, Finland, and with the Neonatal HTSH FEIA PLUS test kit on the NS2400 from Labsystems Diagnostics Oy, Vantaa, Finland. Both test kits were used according to the manufactures instructions.

In total, 5029 normal samples from the routine newborn screening were compared. In addition, 472 samples with initial TSH values between 2 and 460 mU/L on either platform, were repeated in duplicate, on the other platform, respectively, or on both platforms. From these 472 samples, 66 were from newborns with confirmed CH. Furthermore, in total, 36 samples from external quality assessment program (EQA) were measured on the LDx/NS2400 platform. In total, 21 samples came from the german RfB (Reference Institute for Bioanalytics, Bonn, Germany), 11 from UK-NEQUAS (Clinical Chemistry, UK NEQAS Birmingham Quality, Birmingham, UK) and 4 from CDC (CDC, Newborn Screening Quality Assurance Program, Atlanta, GA, USA). The 11 samples from UK-NEQUAS were also measured on the PE/GSP platform. The other EQA samples had, unfortunately, not enough material for parallel measurements.

## 3. Result

### 3.1. Comparison of 5501 Newborn Samples

**Figure 1:**
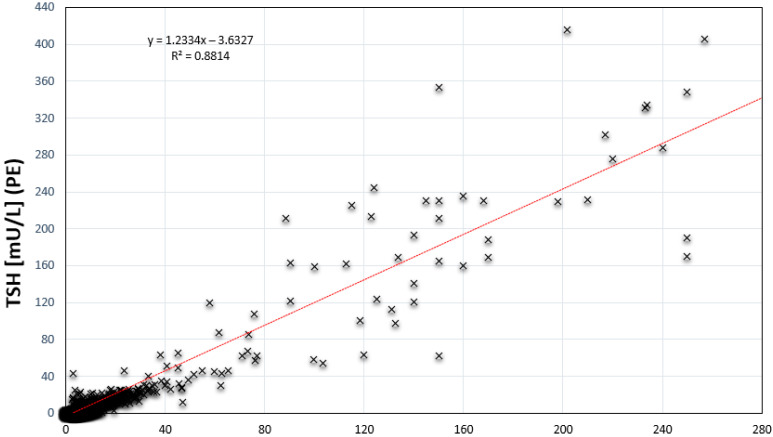
Comparison of TSH determination by Labsystems/NS2400 and PerkinElmer/GSP (*n* = 5029).

For this comparison study 5029 unselected normal samples, 430 samples with TSH > 5 mU/L and 66 samples of newborns with confirmed CH were used. All samples were measured on both platforms with the Labsystems test kits on the NS2400 (LDx/NS2400), and the PerkinElmer test kits on the GSP (PE/GSP). The two assays showed a good correlation with r^2^ = 0.8814 (Figure 1). Comparable correlation coefficients have been published for the comparison of automated serum immunoassays, where r^2^ of 0.849 with a 95% CI of 0.609–0.946 (*p* < 0.001), was considered as “significant relationship” [14].

**Figure 2:**
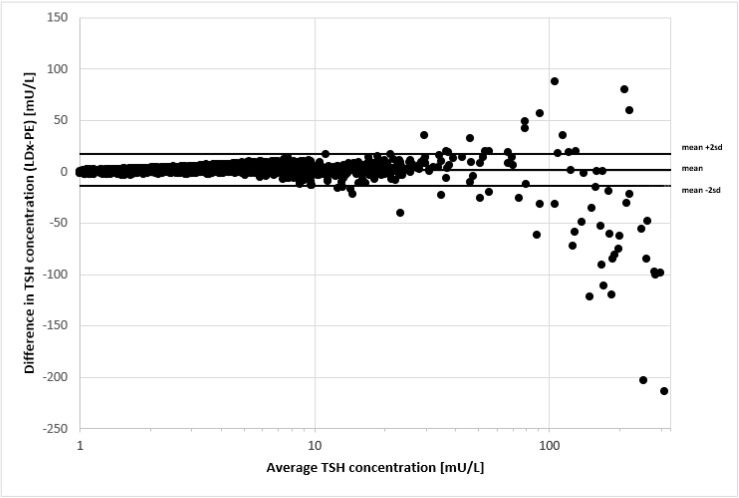
Comparison of TSH determination by Labsystems/NS2400 and PerkinElmer/GSP. The difference of analyte concentration is plotted against the average analyte concentration determined by the two methods (*n* = 5029).

The TSH values were slightly higher with LDx/NS2400, with a mean difference of +2.09 mU/L and a standard deviation of 7.67 mU/L. Figure 2 shows the same samples in a bland-altman plot. There is a very good agreement for TSH values up to 50 mU/L, and only for TSH values > 100 mU/L the variation increases significantly. The same observation was made, when the PerkinElmer AutoDelfia assay was compared to the PerkinElmer GSP assay [12], and also for other serum immunoassays [14]. However, there were no misclassifications of the confirmed positive cases. Furthermore, the variation for TSH values > 100 mU/L in a screening test, has no clinical relevance. The range of TSH concentrations for the 66 cases with confirmed CH was 19.2–267 mU/L on the LDx/NS2400 platform, and 16.1–565 mU/L on the PE/GSP platform. The discrepancy at concentrations > 250 mU/L is explained by the fact that the calculation program of the PE/GSP extrapolates concentration that are higher than the highest calibrator, while the calculation program of the LDx/NS2400 does not, which is in line with GLP, and standard clinical chemistry regulations.

### 3.2. Comparison of 472 Newborn Samples in Duplicate

**Figure 3:**
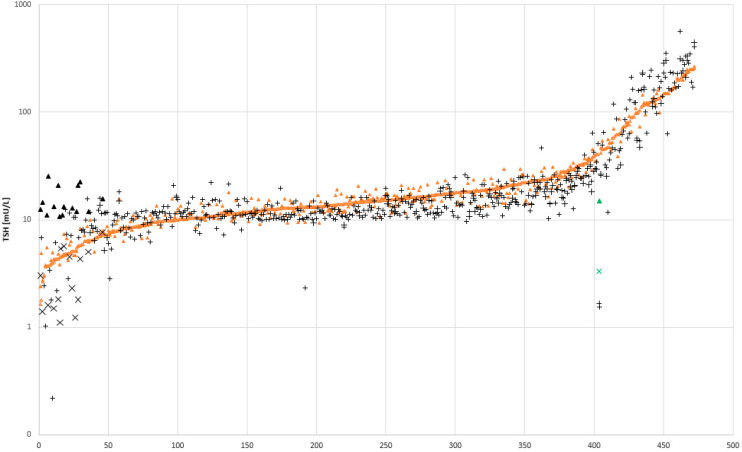
Comparison of 472 samples with TSH values between 2–460 mU/L. All samples were measured in duplicate on both platforms, Labsystems/NS2400 and PerkinElmer/GSP. n = sample number; **♦** measured with LDx, + measured with PE, sample pairs with suggested washer problem: **▲** and **x** measured with LDx, ▲ and **x** measured with PE. Samples were sorted arbitrary, according to increasing TSH values of the first LDx measurement.

During the comparison described in chapter 3.1 we observed some very discrepant results, where elevated TSH concentrations could not be reproduced when the measurement was repeated in duplicate on the same platform. Therefore, we compared 472 newborn samples with TSH values between 2 and 460 mU/L in duplicate on both platforms. In Figure 3 these results are shown. Orange rhombs (**♦**) for LDx/NS2400 and black crosses (+) for the PE/GSP. The results of the 472 samples measured in duplicate on both platforms, are deliberately aligned according to the first measurement on the LDx/NS2400. Therefore, one orange rhomb for sample (n) always overlaps with one orange rhomb from the next sample (n + 1), which gives the impression of a sigmoid line. However, important is, that for each sample there are in total four results plotted on the y-axis (two orange rhombs, and two black crosses). Over the entire range we observed a good agreement between both assays. However, there is one sample pair on the LDx/NS2400 platform (**▲**, **x**), and 15 sample pairs on the PE/GSP platform (▲, x), that showed a big discrepancy between the two measurements (on the same platform). The reason for this discrepancy is most probably a problem with the washer or disc remover. Both test kits are so called sandwich ELISA test, where the labeled antibodies bind to a TSH molecule, and this complex binds to a second antibody that is immobilized in the microtiter plate. Excess labeled antibodies are then removed during disk removing and washing. In case the disk from the sample collection device is not removed, the respective well is insufficiently washed, and remaining excess label, or the floating disk itself [13] will lead to erroneously high readings. Furthermore, fibers from the blood collection devices that can be torn off during punching, might partly block the needles of the plate washer, which has the same effect. Over the whole period of comparison and verification, we had in total 42 cases with the same problem. In total, there were 23 on the PE/GSP platform and 19 on the LDx/NS2400 platform.

### 3.3. Day to Day Reproducibility

**Figure 4:**
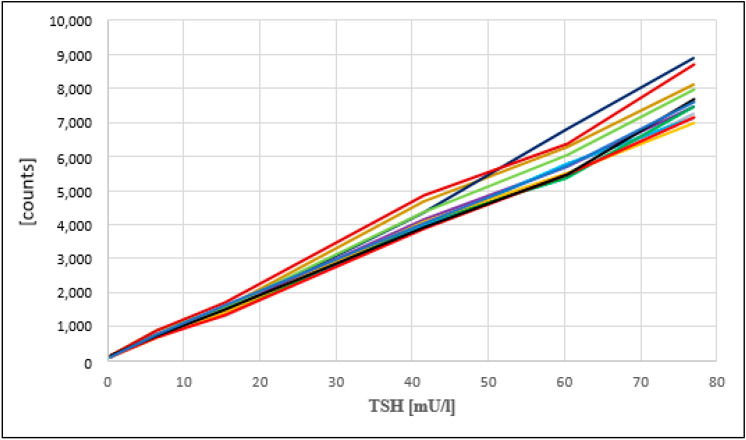
Calibration curves of 12 measurements with the Labsystems/NS2400 over 5 weeks.

Day-to-day reproducibility of the calibration curves for the PE/GSP platform has been described previously [12], with CVs between 5.8 and 8.5 for calibrators A–F. The calibration curves for the LDx/NS2400 platform show the same reproducibility (Figure 4), with CVs between 8 and 13 (Table 1).

### 3.4. Comparison of the Distribution of TSH Values

**Figure 5:**
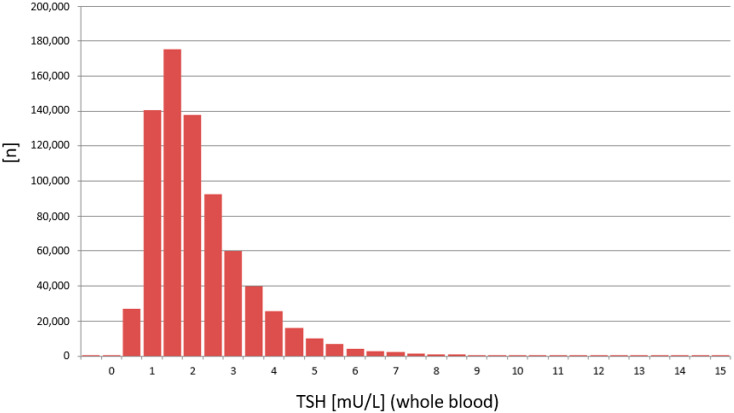
Distribution of TSH values measured on the PE/GSP platform (*n* = 748,180).

**Figure 6:**
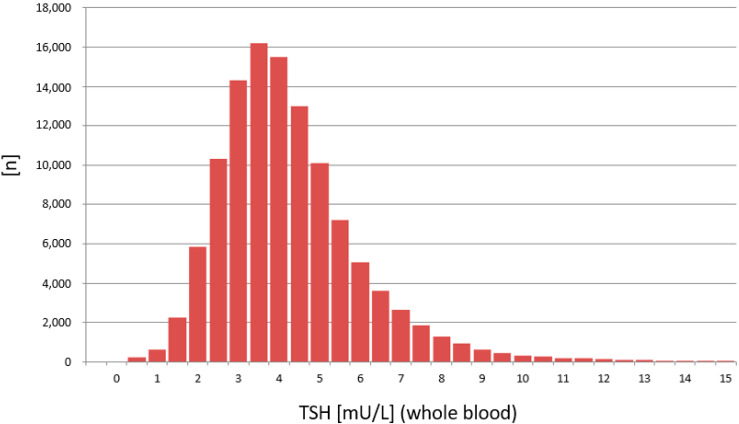
Distribution of TSH values measured on the LDx/NS2400 platform (*n* = 116,260).

The distribution of TSH values was calculated from 748,180 routine newborn samples measured on the PE/GSP platform (Figure 5) and from 116,260 routine newborn samples measured on the LDx/NS2400 platform (Figure 6).

**Figure 7:**
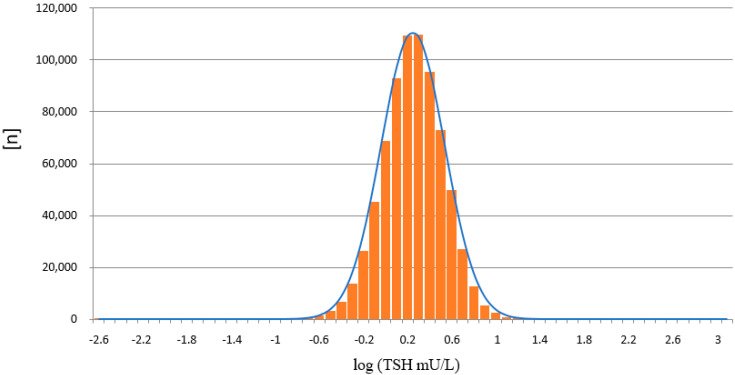
Distribution of TSH values measured on the PE/GSP platform, after logarithmic transformation (*n* = 748,180).

**Figure 8:**
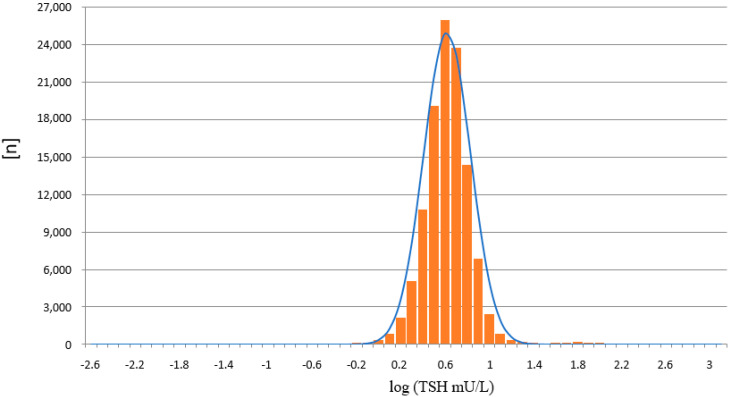
Distribution of TSH values measured on the LDx/NS2400 platform, after logarithmic transformation (*n* = 116,260).

The results from the PE/GSP show a significant right skewed distribution, with a significant number of results with a calculated negative concentration (*n* = 260). Negative concentrations are physically impossible, and should principally not be reported from the calculation software that is part of the PerkinElmer calculation software. For the LDx/NS2400 platform the results are much less right skewed, and the calculation software does not calculate negative concentrations. Since TSH values in the low range are already around or below the limit of quantitation, a logarithmic transformation of the TSH values was made [15], in order to get a better estimation, whether the TSH values are normally distributed or not (Figure 7 and Figure 8). Both assays show a normal distribution. However, the distribution of TSH values measured on the LDx/NS2400 platform was much narrower, than those measured on the PE/GSP platform. This explains the lower recall rate for the LDx/NS2400 platform (0.059%) compared to the PE/GSP platform (0.063%).

### 3.5. Internal and External Quality Control

QC parameters for the PE/GSP platform have also been described previously, with intra assay CVs of 8.9 and 4.9 and inter assay CVs of 8.1 and 8.3, at 15.0 mU/L, and 59.6 mU/L, respectively [12]. For the LDx/NS2400 platform the results are summarized in Table 2. Eleven samples from UK-NEQUAS could be measured in parallel on both platforms.

In addition, 36 samples from EQA schemes were measured on the LDx/NS2400 platform. In total, 21 samples were from RfB, 11 from UK-NEQAS and 4 from CDC (Table 3). QC samples were kept at −18 °C in sealed plastic bags with desiccant, upon arrival in the laboratory. The results of all measured QC samples were within the expected range.

## 4. Discussion

The two platforms for TSH determination from DBS showed a good correlation, and the most important result is that no misclassifications of confirmed cases with congenital hypothyroidism were observed. Neither on the PE/GSP, nor on the LDx/NS2400 platform. The results of the comparison between PE/GSP and LDx/NS2400 was comparable to the comparison of PE/GSP and PE/AutoDelfia [12]. The sample size was nearly the same, 5029 and 4159, respectively, and the Bland–Altman plots also nearly look the same. Variation within ±2 SD from the mean value for samples with TSH up to 50 mU/L, and above 50 mU/L an increasing variation and a shift to one side. However, as already mentioned, this increased variation has no influence on the clinical interpretation. The day to day reproducibility of the counts of the calibration curves were also comparable, with good CVs for both platforms. When we compared the reproducibility of 472 newborn samples, that could be measured in duplicate on both platforms, again both platforms showed very good comparability of the results, apart from 1 sample with discrepant values on the LDx/NS2400 platform, and 15 samples with discrepant values on the PE/GSP platform. During the whole comparison and verification of the LDx/NS2400 platform, we could identify 19 samples with problems with washer or disc remover, while we had 23 cases on the PE/GSP. Here we also see the advantage of the LDx/NS2400 platform. While the PE/GSP is a completely closed system, where the operator has no optical control of the analysis procedures, the LDx/NS2400 is an open system, with the option of intervention. However, these washer/disk remover problems will only cause additional internal repeats, and increase the workload in the laboratory, in cases of non-competitive immune assays, like TSH assays. However, competitive immune assays like 17-OHP assays bear the risk of false negative results, since high fluorescence reads will result in the calculation of a low concentration. The verification of the LDx/NS2400 has revealed the reason for occasionally occurring unreproducible results. During the verification of the LDx/NS2400 platform, we also participated in three different EQA schemes, and results were in the expected range, and the QC scheme was passed. After the positive result of the verification of the LDx/NS2400 platform, the Swiss Newborn Screening laboratory switched to the LDx/NS2400 system. Subsequently we could compare the distribution of TSH values from the swiss cohort measured with both platforms, with a high number of samples. 748,180 measured on the PE/GSP, and 116,260 on the LDx/NS2400 platform. This comparison showed that the distribution of values measured on the PE/GSP were significantly right skewed, while the distribution on the LDx/NS2400 was much less skewed. The logarithmic transformation showed in addition that the distribution of TSH values was much narrower on the LDx/NS2400 platform. However, in the cut-off region (between 10 and 20 mU/L) there was very good correlation, and therefore there was no need to change the reference ranges or cut-offs. In Switzerland we use age dependent cut-offs, <20 mU/L for children 0–48 h and 15 mU/L for children 49–96 h old.

## 5. Conclusions

Both platforms are, without any limitation, suitable for the measurement of TSH from dried blood spots for NBS. TSH values are comparable, and especially in the cut-off range between 10 and 20 mU/L whole blood, there is very good concordance. During the evaluation, and also during routine use of both platforms, no false negative cases occurred. However, due to the narrower distribution of TSH values on the LDx/NS2400 platform, the recall rate, and therefore the number of false positive results was lower.

Apart from the TSH test kit performance, the LDx/NS2400 has one major advantage compared to the PE/GSP. The LDx/NS2400 is an “open” instrument, it can be used also for assays, that use organic solvents, which is strictly inhibited on the closed PE/GSP instrument. Furthermore, all components of the LDx/NS2400 can be used in standalone mode; that means, for example, the washer, disc remover and fluorimeter can also be used for other tests. In addition, those components can also be used during downtime of the complete instrument. This makes the LDx/NS2400 platform much more flexible, and for medium size NBS laboratories it offers the possibility total automation with only one automated platform, instead of also investing directly in a second backup instrument.

## Figures and Tables

**Table 1:** Mean counts and CV of kit calibrators over time, measured on the LDx/NS2400 platform; *n* = 12; mean (CV).

Calibrator A	Calibrator B	Calibrator C	Calibrator D	Calibrator E	Calibrator F
138 (13)	768 (10)	1484 (12)	4190 (8)	5824 (8)	7690 (10)

**Table 2:** Intra- and inter-assay variation of kit controls (C1, C2), and CDC QC samples on the LDx/NS2400 platform.

	Intra-Assay Variation (CV)	Intrer-Assay Variation (CV)	Mean Recovery [%] *
Control 1	6.3 (at 9.7 mU/L; *n* = 10)	15.7 (at 9.7 mU/L; *n* = 14)	103.0
Control 2	8.5 (at 25.1 mU/L; *n* = 10)	6.6 (at 25.1 mU/L; *n* = 14)	
CDC	4.3 (at 18.7 mU/L; *n* = 4)	6.8 (at 45.3 mU/L; *n* = 14)	

* Mean recovery was calculated from all QC samples.

**Table 3:** Results of EQA samples from RfB, UKNEQUAS, and CDC QC samples on the LDx/NS2400 platform; *n* = 36, and EQA samples from UKNEQUAS on the PE/GSP platform; *n* = 11.

Sample	Result [mU/L] PE/GSP	Result [mU/L] LDx/NS2400	Target [mU/L]	Range [mU/L]
RfB-1		2.05	3.22	0.82–5.62
RfB-2		3.53	5.30	2.90–7.70
RfB-3		3.41	5.60	3.20–8.00
RfB-4		7.74	6.90	4.83–8.97
RfB-5		5.78	7.34	4.40–10.30
RfB-6		9.53	8.90	6.23–11.60
RfB-7		6.45	9.05	5.43–12.70
RfB-8		8.99	9.90	6.93–12.90
RfB-9		9.87	12.10	7.26–17.00
RfB-10		9.62	13.00	7.80–18.20
RfB-11		14.52	17.00	10.20–23.80
RfB-12		16.94	18.00	12.60–23.40
RfB-13		13.92	18.80	11.20–26.40
RfB-14		15.12	23.00	13.80–32.20
RfB-15		18.30	24.50	17.10–31.90
RfB-16		25.51	26.50	15.90–37.10
RfB-17		24.19	26.70	18.60–34.80
RfB-18		42.67	33.00	23.10–42.90
RfB-19		23.48	33.30	19.90–46.70
RfB-20		39.52	35.20	24.60–45.80
RfB-21		39.25	51.90	31.10–72.70
UK-NEQUAS-1	3.40	3.33	3.60	1.50–5.70
UK-NEQUAS-2	4.30	4.53	4.60	2.50–6.70
UK-NEQUAS-3	5.90	5.65	5.80	3.40–8.20
UK-NEQUAS-4	11.90	10.27	12.20	7.40–17.00
UK-NEQUAS-5	20.00	15.18	20.40	14.40–26.40
UK-NEQUAS-6	19.90	18.24	21.50	15.80–27.20
UK-NEQUAS-7	30.90	27.10	32.90	22.10–43.70
UK-NEQUAS-8	29.30	28.42	34.20	22.80–45.60
UK-NEQUAS-9	56.30	66.02	66.00	48.00–84.00
UK-NEQUAS-10	69.60	58.91	80.10	56.10–104.10
UK-NEQUAS-11	76.50	65.71	85.00	56.80–113.20
CDC-1		18.53	18.70	11.00–26.30
CDC-2		20.40	22.7	13.40–32.00
CDC-3		49.30	41.50	24.40–58.60
CDC-4		44.67	45.30	26.60–64.00

## Data Availability

The data are not publicly available because of privacy restrictions.
